# Supplementary material for: Molecular dynamics on quantum annealers
Source: Sci Rep. 2022 Oct 7;12:16824. doi: 10.1038/s41598-022-21163-x (PMC9547079; doi:10.1038/s41598-022-21163-x)
Supplement: Supplementary file 3 — Supplementary Information 3. [file 41598_2022_21163_MOESM3_ESM.docx]

Supplementary Materials for

**Molecular Dynamics Simulations on Quantum Annealers**

Igor Gayday, Dmitri Babikov,^*^ Alexander Teplukhin, Brian K. Kendrick, Susan M. Mniszewski, Yu Zhang, Sergei Tretiak, and Pavel A. Dub^*^

*Corresponding author. Email: [dmitri.babikov@mu.edu](mailto:dmitri.babikov@mu.edu)

*Corresponding author. Email: [pdub@lanl.gov](mailto:pdub@lanl.gov)

1. Analytical expressions for potential energy and force fields

The analytical Morse potential energy surface used in this work is given by:

| $V\left( r \right)=D_{e}\left( e^{-2a\left( r-r_{e} \right)}-2e^{-a\left( r-r_{e} \right)} \right)$ | (S  1) |
| --- | --- |

The parameters of this potential were adjusted to represent a hydrogen molecule, namely $D_{e}\approx36450$ cm^-1^, $a\approx1.04$ Bohr^-1^, $r_{e}\approx1.40$ Bohr, where $D_{e}$ is the dissociation energy, $a$ is the Morse constant (steepness of potential), and $r_{e}$ is the equilibrium position (lowest potential energy point). The resulting potential is shown in **Figure S1**.

The force field derived from this potential is given by:

| $F\left( r \right)=-\frac{dV\left( r \right)}{dr}=2aD_{e}\left( e^{-2a\left( r-r_{e} \right)}-e^{-a\left( r-r_{e} \right)} \right)$ | (S  2) |
| --- | --- |

**
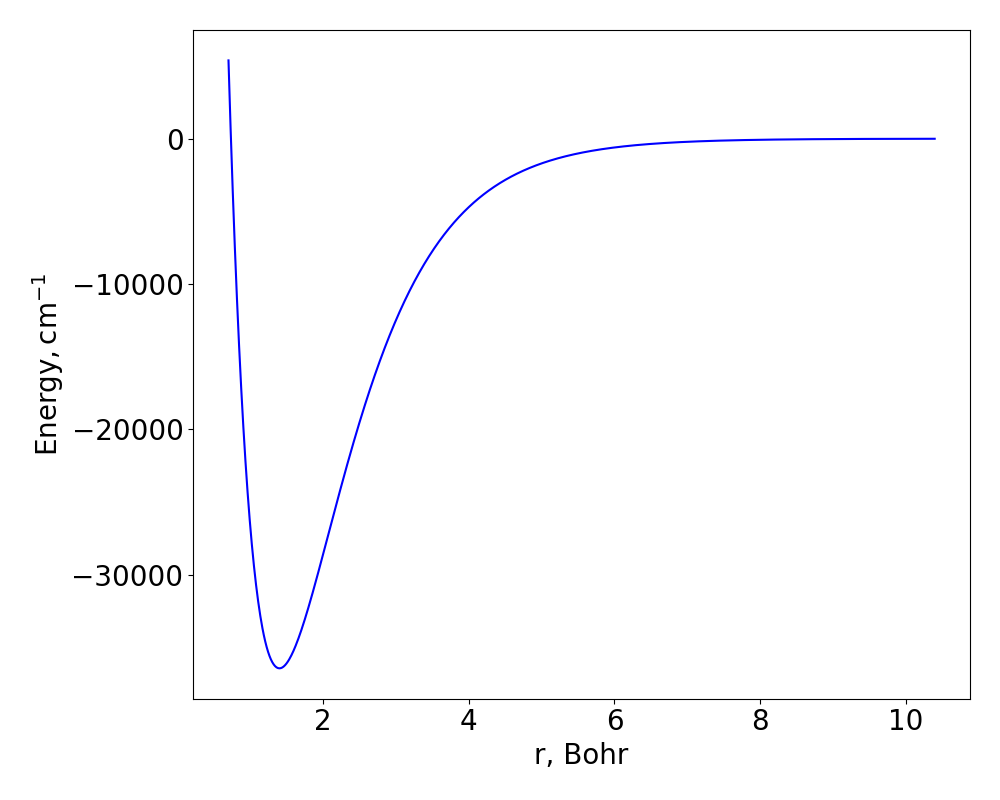
**

**Figure S1.** Potential energy surface used in this work.

1. Analytical solution to equations of motion in a Morse potential

The system of Eq. (1) in the main text can be re-written as a single second order differential equation:

| $\frac{d^{2}r}{dt^{2}}=\frac{F\left( r \right)}{\mu}$ | (S  3) |
| --- | --- |

Analytical solution to Eq. (S3) for initial position $r_{0}$ and zero initial speed can be written as:

| $r\left( t \right)=\frac{1}{a}\ln\left( c_{1}^{2}\tau\frac{c_{3}D_{e}+\left( D_{e}-\frac{c_{4}}{\tau} \right)^{2}}{2c_{1}c_{3}c_{4}} \right)$ | (S  4) |
| --- | --- |

where

| $c_{1}=e^{ar_{e}}$ | (S  5) |
| --- | --- |
| $c_{2}=e^{ar_{0}}$ | (S  6) |
| $c_{3}=-D_{e}\frac{c_{1}}{c_{2}}\left( 2-\frac{c_{1}}{c_{2}} \right)$ | (S  7) |
| $c_{4}=D_{e}+\frac{c_{2}c_{3}}{c_{1}}$ | (S  8) |
| $\tau=e^{\sqrt{\frac{2c_{3}}{\mu}}at}$ | (S  9) |

Note that this equation only provides us with the value of coordinate, so momentum needs to be calculated separately: either numerically (e.g. finite difference) or analytically from conservation of energy.

1. RMSE

The analytical solution of Eq. (S4) was used to calculate RMSE for a given solution $\hat{r}(t)$ as:

| $\mathrm{RMSE}=\sqrt{\frac{1}{M}\sum_{i=1}^{M} \left( \hat{r}_{i}-r_{i} \right)^{2}}$ | (S  10) |
| --- | --- |

1. Performance of greedy with poor D-Wave answers

**Figure** **S2** shows what happens when the quality of D-Wave’s initial guess in the hybrid D-Wave + greedy approach is deliberately lowered by reducing the total number of reads to 10. Comparing these very wrong trajectories with the perfect results of D-Wave + greedy in the main text, where the number of reads was 10000, one can see that the quality of D-Wave’s initial guess is important and D-Wave’s involvement is essential.


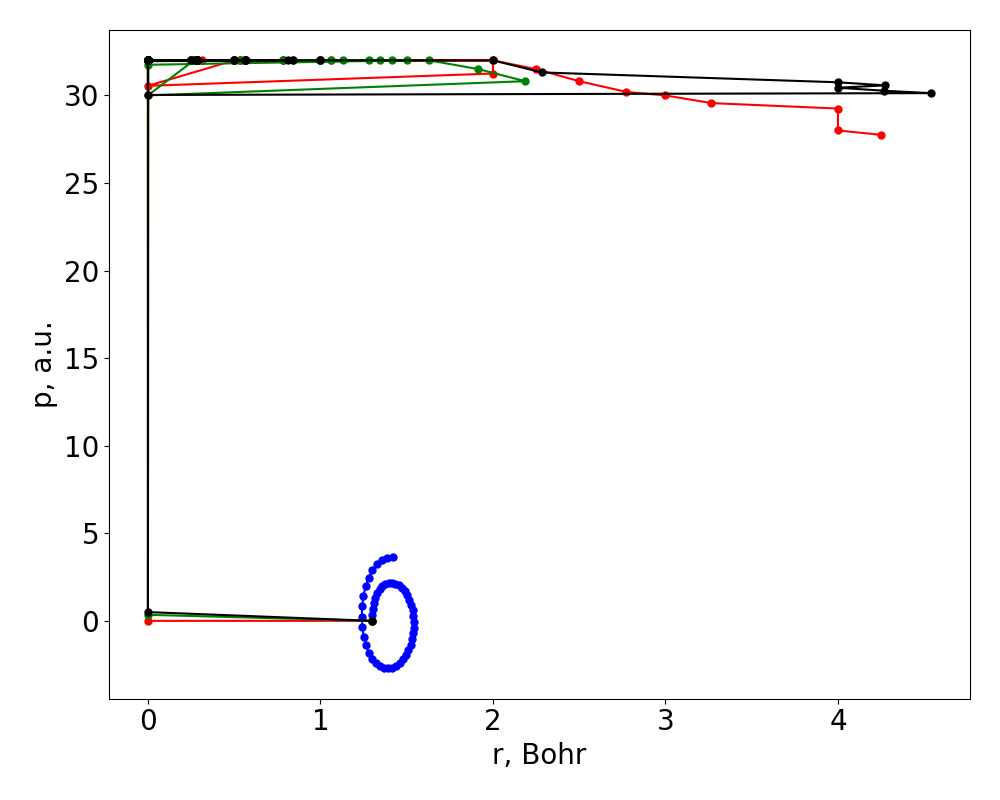


**Figure S2.** Several examples of trajectories obtained with hybrid D-Wave + greedy method, when the quality of D-Wave’s answers was deliberately lowered. The blue line shows ideal QP trajectory for comparison. The remaining three lines are three attempts to solve with D-Wave + greedy.

1. Details of data rescaling

Rescaled results in Figure 4 of the main text were obtained by dividing momentum by 20 and shifting coordinate by -1.4 Bohr.
